# Short Survey on the Protein Modifications in Plasma during SARS-CoV-2 Infection

**DOI:** 10.3390/ijms241814109

**Published:** 2023-09-14

**Authors:** Agnieszka Gęgotek, Neven Zarkovic, Biserka Orehovec, Morana Jaganjac, Suzana Borovic Sunjic, Elżbieta Skrzydlewska

**Affiliations:** 1 Department of Analytical Chemistry, Medical University of Bialystok, Kilinskiego 1, 15-069 Bialystok, Poland; agnieszka.gegotek@umb.edu.pl; 2 Ruder Boskovic Institute, Div. Molecular Medicine Laboratory for Oxidative Stress Bijenicka 54, HR-10000 Zagreb, Croatia; zarkovic@irb.hr (N.Z.); morana.jaganjac@irb.hr (M.J.); suzana.borovic.sunjic@irb.hr (S.B.S.); 3 Clinical Hospital Dubrava, HR-10000 Zagreb, Croatia; biserka.orehovec@gmail.com

**Keywords:** COVID-19, plasma proteome, protein adducts, 4-hydroxynonenal, malondialdehyde, 15-deoxy-12,14-prostaglandin J2

## Abstract

Although the COVID-19 pandemic has ended, it is important to understand the pathology of severe SARS-CoV-2 infection associated with respiratory failure and high mortality. The plasma proteome, including protein modification by lipid peroxidation products in COVID-19 survivors (COVID-19; *n* = 10) and deceased individuals (CovDeath; *n* = 10) was compared in samples collected upon admission to the hospital, when there was no difference in their status, with that of healthy individuals (Ctr; *n* = 10). The obtained results show that COVID-19 development strongly alters the expression of proteins involved in the regulation of exocytosis and platelet degranulation (top 20 altered proteins indicated by analysis of variance; *p*-value (False Discovery Rate) cutoff at 5%). These changes were most pronounced in the CovDeath group. In addition, the levels of 4-hydroxynonenal (4-HNE) adducts increased 2- and 3-fold, whereas malondialdehyde (MDA) adducts increased 7- and 2.5-fold, respectively, in COVID-19 and CovDeath groups. Kinases and proinflammatory proteins were particularly affected by these modifications. Protein adducts with 15-deoxy-12,14-prostaglandin J2 (15d-PGJ2) were increased 2.5-fold in COVID-19 patients, including modifications of proteins such as p53 and STAT3, whereas CovDeath showed a decrease of approximately 60% compared with Ctr. This study for the first time demonstrates the formation of lipid metabolism products—protein adducts in plasma from survived and deceased COVID-19 patients, significantly distinguishing them, which may be a predictor of the course of SARS-CoV-2 infection.

## 1. Introduction

The pandemics of COVID-19 caused by infection with the SARS-CoV-2 coronavirus have affected the entire world. According to official data from the World Health Organization (accessed on 26 June 2023), about seven million people died from this disease within four years (2019–2023) [1]. The early symptoms of SARS-CoV-2 infection resembled those of influenza infection. Many patients suffered from respiratory failure leading to interstitial changes in the lungs. However, in severe cases, renal failure, coagulation disorders, and sepsis occurred, which were the cause of the high mortality rate of the disease [2].

**Figure 1:**
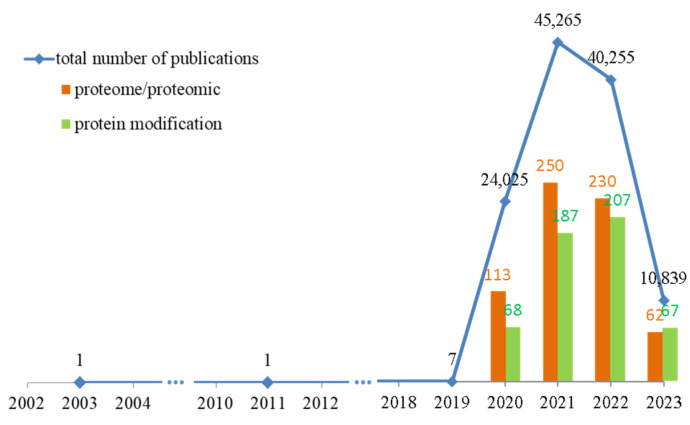
The number of published studies on changes in patients’ bodies caused by SARS-CoV-2 infection over the past 10 years, with emphasis on proteomics studies and articles containing analyses of protein modifications. Data obtained from Web of Science Core Collection (accessed on 26 June 2023) [3].

Therefore, the pathophysiology of COVID-19 has been extensively studied. Data from the Web of Science Core Collection (accessed on 26 June 2023) show that more than 120,000 articles have been published addressing the changes caused by SARS-CoV-2 infection (Figure 1) [3]. However, less than 1% of these studies used a comprehensive proteomics approach to identify specific biomarkers for this disease or molecules that could predict the course of infection.

Previously, it has been described that the development of COVID-19 is also accompanied with a significant disturbance of redox homeostasis resulting in a high level of oxidative stress that is related to the severity of the damage caused by the infection [4,5]. The decrease in the activity of antioxidant enzymes (superoxide dismutases 1 and 2) and the decrease in the amount of low-molecular-weight antioxidants (glutathione, vitamins A and E) observed in the patient’s plasma [4] also favors pro-oxidant metabolism in the patient’s body. As a consequence of COVID-19, increased lipid metabolism has been observed, due to both oxidative stress and increased activity of phospholipase A2 (PLA2), lipoxygenases (LOXs), and cyclooxygenases (COXs) [5,6], manifested by an increased level of lipid metabolism products, including reactive aldehydes, such as 4-hydroxynonenal (4-HNE) and malondialdehyde (MDA), and cyclization products like neuroprostanes/isoprostanes and prostaglandins [4,5,7].

The above-mentioned lipid peroxidation products have been identified as signaling agents in the promotion of proinflammatory pathways, including activation of the NFκB factor [8,9,10,11]. In addition, lipid-peroxidation-derived reactive aldehydes can bind to the nucleophilic amino acid side chain of proteins modifying protein structure and function, which is true for both proinflammatory proteins and proteins related to antioxidant response or apoptosis [12]. To date, however, little is known about the modification of proteins by lipid peroxidation products that have occurred in the bodies of COVID-19 patients. Of particular importance seem to be data correlating the degree of protein modification by 4-HNE with the severity and impact of SARS-CoV-2 infection, which may even lead to death [7]. MDA adducts with the innate immune system protein (protein D) are also equally important for neutralizing the SARS-CoV-2 virus [13]. These data already show how important protein modifications by products of lipid metabolism are for the course of infection and how important they can be for the treatment process. Therefore, the aim of this study was to analyze the proteomic profile of plasma samples derived from deceased and surviving COVID-19 patients with particular reference to the rate of formation and biological efficacy of proteins modified by products of lipid metabolism, such as reactive aldehydes 4-HNE and MDA, as well as 15-deoxy-12,14-prostaglandin J2 (15d-PGJ2), in comparison with healthy individuals.

## 2. Results

**Figure 2:**
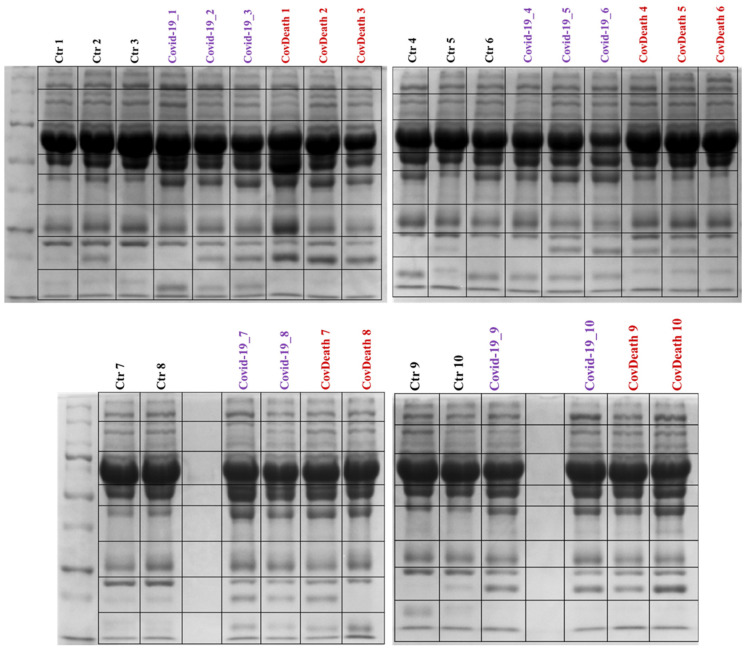
Electrophoretic separation and staining with Coomassie Brilliant Blue R-250 of plasma proteins from healthy donors (Ctr, *n* = 10) and COVID-19 patients (COVID-19—surviving patients, *n* = 10, and CovDeath—deceased patients, *n* = 10). The lines of the black frames indicate how the gels were cut before protein digestion.

Proteomics analyses revealed differences in protein expression among the three study groups: COVID-19 survivors (COVID-19), COVID-19 deceased patients (CovDeath), and healthy donors who were set as the control group (Ctr). Below are described point by point the most important statistically significant results, including those resulting from gel profiling, PCA statistical analysis, as well as levels of individual top-altered proteins and their modifications by lipid peroxidation products. Differences between all studied groups were already observed during sample preparation, as the lower parts of the SDS-PAGE gels varied in the distribution of bands between samples (Figure 2).

A comprehensive proteomics workflow allowed the identification and label-free quantification of 1477 proteins in all analyzed samples that met the established cut-off parameters, as described in methodological section (Supplementary file T1).

**Figure 3:**
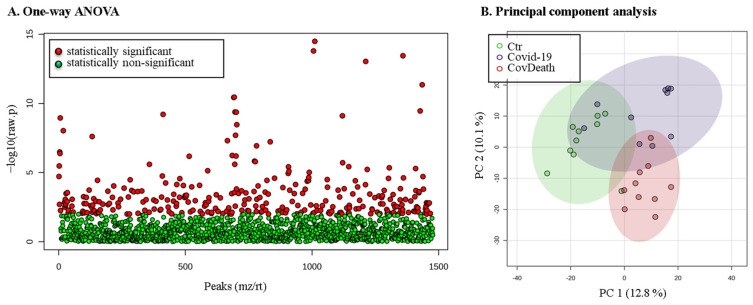
The results of statistical analysis of protein level quantified in plasma from healthy donors (Ctr, *n* = 10), COVID-19 patients (COVID-19—surviving patients, *n* = 10), and CovDeath (COVID-19 deceased patients, *n* = 10). (**A**) One-way ANOVA showing p distribution within all data and (**B**) results of principal-component analysis (PCA).

The expression of 299 of these proteins was found to be significantly altered (Figure 3A). PCA showed distinct proteomic signatures for the Ctr and CovDeath groups, whereas the COVID-19 survivor group showed partial overlap with the Ctr or CovDeath groups, despite the strong concentration of samples in the upper-right corner of the plot, (Figure 3B).

**Figure 4:**
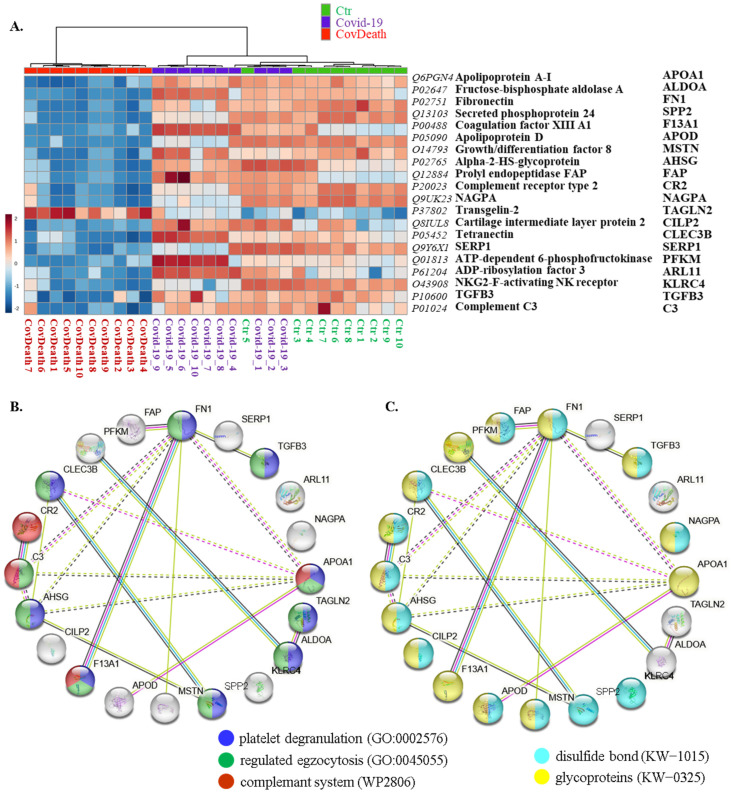
(**A**) Heatmap and clustering for the top 20 altered proteins from the plasma from healthy donors (Ctr, *n* = 10), COVID-19 patients (COVID-19—surviving patients, *n* = 10), and CovDeath (COVID-19 deceased patients, *n* = 10). Protein expression levels (log transformed) were scaled to the row mean. The color key relates the heatmap colors to the standard score (z-score), which is the deviation from the row mean in units of standard deviation above or below the mean. (**B**) The main biological pathways in which proteins selected in the heatmap participate and (**C**) structural modifications of these proteins. Data analyzed using STRING.11.5. Keywords used (KW): (KW-0325) protein containing one or more covalently linked carbohydrates of various types, i.e., from monosaccharides to branched polysaccharides, including glycosylphosphatidylinositol-inositol (GPI) and glycosaminoglycans (GAG); (KW-1015) protein which is modified by the formation of a bond between the thiol groups of two peptidyl-cysteine residues.

This distribution was consistent with the clustering of samples in the case of the top twenty modified proteins shown in the heatmap (Figure 4A). For half of these proteins, the main function was defined as regulation of exocytosis (10/20) and platelet degranulation (9/20), including fibronectin (FN1, P02751), transforming growth factor β-3 proprotein (TGFB3, P10600), apolipoprotein A-I (APOA1, Q6PGN4), transgelin-2 (TAGLN2, P37802), fructose-bisphosphate aldolase A (ALDOA, P02647), growth/differentiation factor 8 (MSTN, O14793), α2-HS-glycoprotein (AHSG, P02765), coagulation factor XIII A1 (F13A1, P00488), complement C3 (C3, P01024), and tetranectin (CLEC3B, P05452) (Figure 4B). Moreover, 70% of them were found to be glycoproteins (KW-0325) (14/20) and 65% of them were modified by the formation of a bond between the thiol groups of two peptidyl-cysteine residues (KW-1015) (13/20) (Figure 4C).

**Figure 5:**
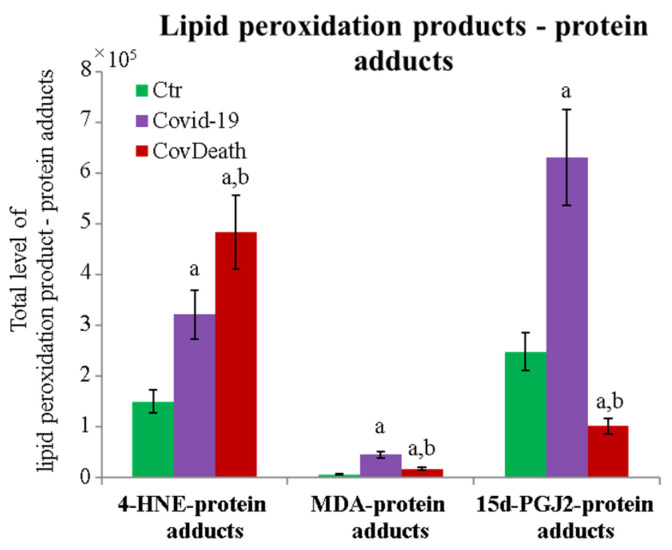
Total level of protein modifications by lipid peroxidation products 4-hydroxynonenal (4-HNE), malondialdehyde (MDA), and 15-deoxy-12,14-prostaglandin J2 (15d-PGJ2) in plasma from healthy donors (Ctr, *n* = 10), COVID-19 patients (COVID-19—surviving patients, *n* = 10), and CovDeath (COVID-19 deceased patients, *n* = 10). The levels of lipid peroxidation product–protein adducts were estimated based on the peak intensity of modified peptides. Mean values ± SD are presented. ^a^ statistically significant differences vs. Ctr group, *p* < 0.05; ^b^ statistically significant differences vs. COVID-19 group, *p* < 0.05.

The protein expression profile was not the only factor that distinguished the analyzed groups. The degree of protein modification by lipid peroxidation also varied considerably among the different study groups. A significant increase in the level of proteins modified by reactive aldehydes was observed (Figure 5). In plasma from COVID-19 patients, 4-HNE–protein adducts increased more than two-fold and more than three-fold in plasma from patients who did not survive because of infection.

**Figure 6:**
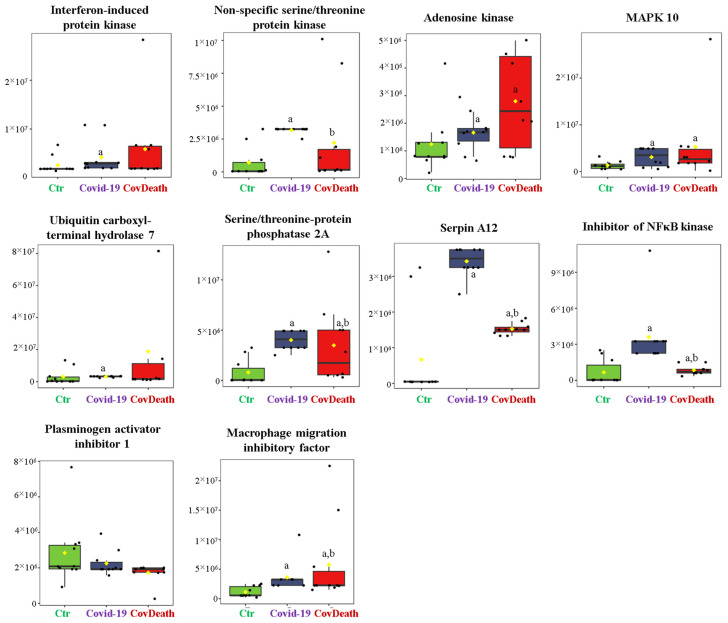
The level of 4-hydroxynonenal (4-HNE)–protein adducts in plasma from healthy donors (Ctr, *n* = 10), COVID-19 patients (COVID-19—surviving patients, *n* = 10), and CovDeath (COVID-19 deceased patients, *n* = 10). The levels of lipid peroxidation product–protein adducts were estimated based on the peak intensity of modified peptides. ^a^ statistically significant differences vs. Ctr group, *p* < 0.05; ^b^ statistically significant differences vs. COVID-19 group, *p* < 0.05.

**Figure 7:**
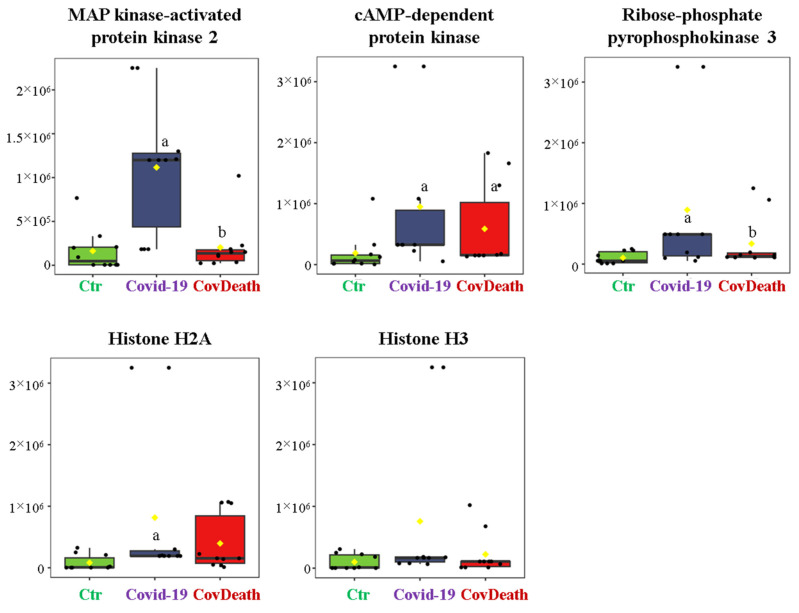
The level of malondialdehyde (MDA)–protein adducts in plasma from healthy donors (Ctr, *n* = 10), COVID-19 patients (COVID-19—surviving patients, *n* = 10), and CovDeath (COVID-19 deceased patients, *n* = 10). The levels of lipid peroxidation product–protein adducts were estimated based on the peak intensity of modified peptides. ^a^ statistically significant differences vs. Ctr group, *p* < 0.05; ^b^ statistically significant differences vs. COVID-19 group, *p* < 0.05.

**Figure 8:**
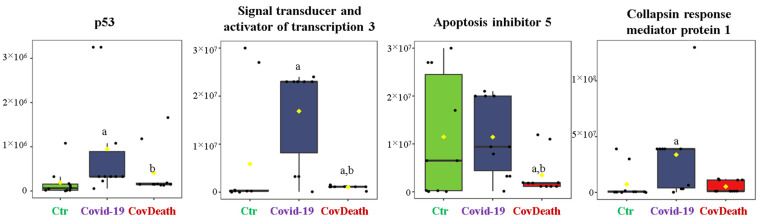
The level of 15-deoxy-12,14-prostaglandin J2 (15d-PGJ2)–protein adducts in plasma from healthy donors (Ctr, *n* = 10), COVID-19 patients (COVID-19—surviving patients, *n* = 10), and CovDeath (COVID-19 deceased patients, *n* = 10). The levels of lipid peroxidation product–protein adducts were estimated based on the peak intensity of modified peptides. ^a^ statistically significant differences vs. Ctr group, *p* < 0.05; ^b^ statistically significant differences vs. COVID-19 group, *p* < 0.05.

The dominant 4-HNE-modified proteins were serpin A12 (Q8IW75), inhibitor of NF-κB kinase (O15111), macrophage migration inhibitory factor (P14174), and kinases: adenosine kinase (P55263), MAPK 10 (P53779), and non-specific serine/threonine protein kinase (Q9Y6B5). All proteins modified by 4-HNE are shown in Figure 6. For protein adducts with MDA, the highest level was observed in COVID-19 surviving patients, which was seven times higher than the Ctr group. In the CovDeath group, the level of MDA–protein adducts was about 2.5 times higher than the Ctr group. This result was related to the increased levels of MDA adducts with kinases (cAMP-dependent protein kinase (P22612), ribose-phosphate pyrophosphokinase 3 (P21108), and MAP kinase-activated protein kinase 2 (P49137)), which were mainly observed in COVID-19 surviving patients. Histones H2A (Q8IUE6) and H3 (Q5TEC6) were also altered to an increased extent (Figure 7). Interestingly, although a 2.5-fold increase in 15d-PGJ2 protein adducts was observed in the COVID-19 surviving group, a decrease of up to 60% was observed in the COVID-19 deceased group (Figure 5). This was particularly true for proteins such as p53 (S5LQU3), collapsin response mediator protein 1 (CRMP1, X5DNI1), signal transducer and activator of transcription 3 (STAT 3, P40763), and apoptosis inhibitor 5 (API5, Q9BZZ5) (Figure 8).

## 3. Discussion

Since the COVID-19 pandemic has dominated the modern world, the researchers are putting joint efforts to understand the pathogenesis of this disease. The fact that COVID-19 patients can develop pneumonia, severe symptoms of acute respiratory distress syndrome, and multiple-organ failure connected with post-acute conditions, further encourages an in-depth analysis of this disease [14,15]. The results obtained in this study show that COVID-19 development strongly alters the expression of proteins involved in the regulation of exocytosis and platelet degranulation, simultaneously increasing 4-HNE/MDA–protein adducts, thus affecting the structure of kinases and proinflammatory proteins. In addition, in the plasma of COVID-19 patients who survived and those who died, different changes (increase and decrease, respectively) were found for the protein adducts with 15d-PGJ2. It has been described previously that infection with COVID-19 triggers a systemic immune response in the human body, manifested by increased expression of proinflammatory cytokines, including interleukins (IL-1, 6, 8) and tumor necrosis factor α (TNFα) [16,17,18,19]. However, it remains unclear whether some of the proinflammatory proteins are specific to COVID-19 and how signaling pathways are involved in the progression of infection [20,21]. In this study, the whole proteome of plasma from COVID-19 patients was analyzed, with particular attention to metabolic pathways associated with different responses between survivors and deceased patients, as well as differences in the interaction of protein and lipid peroxidation products in the study groups.

### 3.1. COVID-19-Induced Changes in Protein Profile

The results of this study clearly show that half of the twenty major proteins whose expression is altered in the plasma of COVID-19 patients are involved in the regulation of exocytosis and platelet degranulation. These observations are in agreement with data from the literature, and both processes enable the spread of the virus in the body [22,23]. After replication in lysosomes, the virus requires an extracellular egress and simultaneously blocks autophagy [24]. This is the mechanism by which SARS-CoV-2 interacts with host proteins to promote its spread. Moreover, the previously described changes in exocytosis, which regulates protein expression, are closely associated with the severity of COVID-19 [23], which is also observed between survivors and deceased patients in our study. At the same time, the activation of platelets stimulates the host immune system to fight SARS-CoV-2 infection [25]. Platelets contain multiple receptors that interact with specific ligands, including proteins of pathogens [26]. These are receptors such as Toll-like receptors (TLRs) or the family of C-type lectin receptors, but also glycoproteins that allow pathogens to interact with platelets [27]. The aforementioned receptors are considered important components in the activation of the innate immune response against SARS-CoV-2 [28]. Moreover, platelet activation or degranulation during COVID-19 may promote vascular and coronary thrombosis [28]. It was found that platelets in severely ill COVID-19 patients were much more hyperactivated than those in mildly ill patients [29,30]. Moreover, the results of this study indicated that in the patients with the worst prognosis, most of the proteins responsible for platelet degranulation are silenced, suggesting that the body capitulates and stops making an effort to eliminate the pathogens. However, this observation was not true for transgelin-2 (TAGLN2, P37802), whose levels were highest in the CovDeath group. The biological roles of transgelin-2 are not fully understood, but it is known that it is involved in apoptosis inhibition, induction of cell proliferation, and promotion of cancer metastasis, corresponding to stimulation of the expression of ABC transporters and PI3K/Akt kinase activity [31]. In the lung, transgelin-2 has been found to be involved in reducing pulmonary resistance in asthma by airway muscle cell relaxation [32]. Thus, it is possible that transgelin-2 has a significant role in lung function in COVID-19 patients. However, transgelin-2 cannot be a COVID-19 prognostic biomarker because its high levels are also associated with other diseases such as asthma, type 2 diabetes, or cancer [33,34,35].

The function and activity of individual proteins are influenced not only by their concentration but also by all post-translational modifications. It is well known that during the development of the disease, various pathological conditions, including oxidative stress and inflammation, affect the protein structure and function of signaling molecules and enzymatic proteins by modifying their active centers [36,37]. The obtained results show that within the top twenty altered proteins, more than half are modified by the formation of a bond between the thiol groups of two peptidyl-cysteine residues. This is certainly related to the oxidative stress found in COVID-19 patients [4,38]. The formation of double bonds between cysteine residues within or between proteins is due to the decrease in the amount of reduced thiol group donors, including glutathione and thioredoxin, but also to increased activity of enzymes (glutathione peroxidase and thioredoxin reductase) that also use them as cofactors [4]. In addition, the proportion of proteins forming double bonds is lower in the CovDeath group compared to the survivors, which also corresponds to smaller changes in antioxidant system levels/activities compared to the control group [4]. This may suggest that the degree of protein modification may be a predictor of disease progression.

### 3.2. Protein Modifications by Reactive Aldehydes in COVID-19 Patients

Reactive aldehydes such as 4-HNE or MDA are the main known products of lipid peroxidation with the described role of signaling molecules both in health and disease [39]. The rate of increase of these molecules determines whether cells will survive by activating their antioxidant system or whether they induce mutation or apoptosis [39]. In the plasma of COVID-19 patients, a strong increase in the activity of enzymes responsible for lipid metabolism, such as phospholipase A2, is one of the factors explaining the increase in lipid peroxidation products, including the aforementioned reactive aldehydes [5]. As a result, both 4-HNE and MDA can directly interact with proteins, altering their structure and function. In general, such adducts support the antioxidant response through changes in transcriptional activity or stimulate proinflammatory signaling, although interactions with particular proteins can lead to specific effects [12].

The amount of the 4-HNE-modified proteins in the plasma of COVID-19 patients compared with the other protein modifications analyzed confirmed our previous findings suggesting that 4-HNE may be directly involved in the pathogenesis of COVID-19, especially with a lethal outcome [7,11,40]. Namely, the 4-HNE-modified proteins were first detected in the kidneys of the deceased COVID-19 patient [38]. Further study involving severely ill COVID-19 patients revealed higher levels of the 4-HNE-modified proteins upon admission to the hospital in the plasma of patients who died after five days than were the levels of the 4-HNE–protein adducts in plasma of survivors [7]. Moreover, dynamic changes of the 4-HNE–protein adducts in plasma indicating a kind of systemic oxidative stress response to the SARS-CoV-2 infection were observed upon admission only for patients who recovered, being absent in the plasma of patients who eventually passed away [7]. Preliminary immunohistochemical findings conducted for the 4-HNE–protein adducts in the lungs of one deceased patient, applying the same monoclonal antibody specific to the 4-HNE–histidine adducts used for the ELISA detection of the 4-HNE–protein adducts in plasma, suggested that 4-HNE might be associated with the fatal outcome. That was later confirmed in another study by the post-mortem immunohistochemical evaluation of the vital organs of the additional nine patients [11].

It is likely that modification of proteins by 4-HNE might occur during the process of death, causing their abundance in the organs of the dead patients, but in spite of that, their distribution was not random and diffuse, as inflammatory cells were immunohistochemically negative, while blood vessels and their content, as well as edematous liquid, were strongly positive for 4-HNE [11,38]. It is also certain that 4-HNE–protein adducts were increased at the time of admission to the hospital in the plasma of patients who eventually died when there was no difference in any relevant parameter between these patients and survivors [7]. Therefore, we assume that modification of proteins by 4-HNE in patients with COVID-19 might be relevant for the pathogenesis of the disease and its final outcome, at least in severely ill patients.

In the current study of plasma samples collected upon admission to the hospital, when there was no difference between the status of survivors and patients who eventually passed away revealed that the 4-HNE modifications mainly affected proteins involved in immune response, including serpin A12 (Q8IW75), inhibitor of NF-κB kinase (O15111), and macrophage migration inhibitory factor (MIF, P14174). Serpins are potent serine protease inhibitors with potential anti-inflammatory properties [41]. It is known that their oxidative modifications lead to their inactivation [42]; therefore, the formation of adducts with 4-HNE should also suppress their anti-inflammatory effect. At the same time, 4-HNE activates the NF-κB-dependent proinflammatory signaling pathway [43], also by interacting with the inhibitor of NF-κB kinase as well as with other kinases important for proinflammatory signaling. Unfortunately, there are no data in the literature on how such modification affects the functioning of MIF. MIF is a cytokine whose activity arrests immune cell movement [44], thereby blocking the inflammatory response. Moreover, the isoform of MIF has been described as an important determinant of COVID-19 symptomatic infection and severity [42,43]. Therefore, further attention should be paid to this protein and the modification of its structure depending on the isoform, which would improve the prognosis of disease progression.

To date, little is known about the activity of specific proteins modified by MDA. In the plasma of the COVID-19 patients studied, a large group of modified MDA proteins are kinases (MAP kinase-activated protein kinase 2 (MAPKAPK2, P49137), ribose-phosphate pyrophosphokinase 3 (PRPS1L1, P21108), and cAMP-dependent protein kinase (PKA, P22612)). Some kinases are known to be activated when modified with MDA [12]. For example, in SARS-CoV-infected Vero E6 cells, MAPKAPK2 is activated resulting in p38/MAPK pathway stimulation leading to the upregulation of Bax expression, which in turn triggers apoptosis and severely damages lung tissue [45,46]. Therefore, additional MDA-induced kinase upregulation further worsens the health of patients. Moreover, transcriptomic analysis of SARS-CoV-2 infection highlighted a correlation between the host-associated signaling pathway and the virus based on the PKA-dependent pathway. In cells in which this pathway was knocked down using small interfering RNAs, replication of SARS-CoV-2 genetic material was inhibited by approximately 50% [47]. However, in COVID-19 patients, histones H2A (Q8IUE6) and H3 (Q5TEC6) were also modified by MDA to an increased extent. Extracellular (plasma) histones are nuclear proteins that can be released into the extracellular space during apoptosis, necrosis, or netosis formation. Under oxidative stress associated with inflammation and disease development, histones stimulate the release of prototypical sepsis cytokines and decreased cell integrity [48]. Therefore, it has been described that plasma histones are significantly increased in COVID-19 patients [49]. Moreover, the formation of MDA adducts with histones promotes their activity, e.g., by increasing their binding (DNA or protein) properties [50]. Combining these facts with the observed increase in MDA–histone adducts in COVID-19 patients, which was particularly high in those who did not survive the infection, such changes could be an additional prognostic factor in the course of disease development.

### 3.3. Formation of Prostaglandin–Protein Adducts in Plasma of COVID-19 Patients

Prostaglandins (PGs) are the products of enzymatic PUFA metabolism with very broad signaling functions, including the regulation of inflammation and pain sensation [51]. PGJ2, among other PGs, is characterized by a cyclopentenone ring with reactive α,β-unsaturated carbonyl groups that can form adducts with cysteine residues in proteins [52]. Some of the PGJ2, including 15d-PGJ2, have anti-inflammatory and antiviral effects, which is being attempted to be used in COVID-19 therapies [53]. This may be due to the fact that a significant increase in 15d-PGJ2 levels is observed in the plasma of patients with mild COVID-19, whereas no such observation has been made in severe COVID-19 patients who consequently do not survive SARS-CoV-2 infection [5]. The results obtained in this study confirm this, as the amount of 15d-PGJ2-modified proteins is highest in COVID-19 survivors, whereas in deceased COVID-19 patients, the amount of 15d-PGJ2-modified proteins is decreased. This has been demonstrated for p53 and STAT3, which are both factors involved in proliferation, apoptosis, but also inflammation and carcinogenesis [54,55]. Moreover, both proteins are inactivated after modification with 15d-PGJ2 [56,57]. Earlier studies have found STAT3 to be increased in COVID-19 patients [58]; therefore, its modification by 15d-PGJ2 counteracts this change and possibly could lead to restoring homeostasis in COVID-19 survivors. However, since the interaction between SARS-CoV-2 and p53 is not clear [59], further research is needed to understand the biological relevance of 15d-PGJ2–p53 adducts in plasma of COVID-19 patients.

### 3.4. Limitations

The results obtained in this study are subject to certain limitations. Due to the use of non-target proteomic analysis, the number of studied samples was limited to a small representative group of patients, who could be at different stages of the disease, which could not be determined by classical diagnostic methods. Moreover, it is not possible to distinguish if proteome changes, notably protein modifications by the lipid peroxidation products, observed in plasma of decreased patients were caused by SARS-CoV-2 infection, the reaction of the organism to it in the period of at least five days before the death, or by the dying process. Additionally, in the case of some unstable lipid peroxidation product–protein adducts, the sample preparation used for proteomic analysis may mean that some of the complexes were not detected in this analysis.

## 4. Materials and Methods

### 4.1. Samples Collection

Plasma samples were collected upon admission to the hospital from a group of 10 COVID-19 survivors (6 females and 4 males) with a mean age of 62 (54–70) years and from 10 later-deceased patients with COVID-19 (6 females and 4 males) with a mean age of 68 (62–77) years who were treated during the period December 2020 to February 2022 at the Clinical Hospital Dubrava in Zagreb, which served as a national COVID-19 center. Patients were randomly selected from a group of 66 (COVID-19 survivors) and 22 (COVID-19 deceased) patients, whose results of classical analysis have previously been published [4,5]. The number of patients in each group was selected as 10 to provide convergent apparatus conditions to all injections and to ensure high-statistical-power analysis [60]. The control group consisted of ten healthy donors (6 females and 4 males) with a mean age of 43 (30–56) years. The study was conducted in accordance with the Declaration of Helsinki, according to the approval of the ethics committee 2020-1012-13 of the Dubrava Clinical Hospital in Zagreb and written informed consent was obtained from all participants.

Blood samples were collected in ethylenediaminetetraacetic acid (EDTA) tubes and centrifuged at 3000 g for 20 min to separate plasma. The antioxidant butylhydroxytoluene was added to the plasma samples, which were stored at −80 °C until analysis.

### 4.2. Protein Separation and Digestion

Total protein concentration was measured by Bradford assay [61] and 30 μg of proteins was mixed with sample loading buffer (Laemmle buffer containing 5% 2-mercaptoethanol), heated at 95 °C for 7 min, and separated on 12% Tris-Glycine SDS-PAGE gels. Following electrophoresis, gels were fixed in 40% methanol and 10% acetic acid for 1 h and stained with Coomassie Brilliant Blue R-250 for 4 h. Complete lanes were excised from the gel and sliced into eight sections (Figure 2). Each band in the gel was reduced with 10 mM 1,4-dithiothreitol (DTT) and alkylated with 50 mM iodoacetamide (IAA). Samples were then in-gel digested overnight at 37 °C with trypsin (Promega, Madison, WI, USA) at a ratio of 1:50 (trypsin:proteins). Digestion was stopped by the addition of 10% formic acid (FA) in an amount to ensure a final concentration in the samples of 0.1% [62]. The obtained peptide mixture was collected, dried under inert gas, and frozen until analysis.

### 4.3. Proteomic Analysis and Protein Identification

Dried peptides were reconstituted in 5% acetonitrile (ACN) with 0.1% FA and separated using a high-performance liquid chromatography system (Ultimate 3000; Dionex, Idstein, Germany) on a 150 mm × 75 mm PepMap RSLC capillary analytical C18 column with 2 μm particle size (Dionex, LC Packings) at a constant flow rate of 0.300 µL/min. Solvents used for separation were solvent A (5% ACN with 0.1% (*v*/*v*) FA) and solvent B (90% ACN with 0.1% (*v*/*v*) FA), and the separation gradient was set to increase from 5% to 60% B over 55 min. The Q Exactive HF mass spectrometer with an electrospray ionization source (ESI) (Thermo Fisher Scientific, Bremen, Germany) was used to analyze the eluted peptides. The conditions for the analysis for peptide identification have been described in detail previously [63].

Raw data were searched against the UniProtKB-SwissProt database (taxonomy: Homo sapiens, release February 2023) using Proteome Discoverer 2.0 (Thermo Fisher Scientific, Seattle, WA, USA). Peptide mass tolerance was set to 10 ppm, MS/MS mass tolerance was set to 0.02 Da, and up to two missed cleavages were allowed. Dynamic modifications were set as cysteine carbamidomethylation/carboxymethylation, methionine oxidation, and lipid peroxidation products (4-HNE, MDA, 15d-PGJ2) with cysteine/lysine/histidine adducts [64,65]. Label-free quantification of proteins was based on the signal intensities of precursor ions, and the amount of lipid peroxidation product–protein adducts was estimated based on the peak intensity of peptides modified by 4-HNE, MDA, or 15d-PGJ2. Only proteins with at least three identified peptides longer than six amino acid residues and at least two unique peptides were selected for further analysis.

### 4.4. Statistical Analysis

The label-free quantification results of each protein using the open-source MetaboAnalyst 5.0 software (http://www.metaboanalyst.ca (accessed on 1 April 2023)) [66] were log-transformed, auto-scaled (mean-centered and divided by the standard deviation of each variable), and normalized by the median of the protein intensities obtained for each sample, ensuring normal distribution of the samples [67]. All missing values (a total of 12 (<0%) missing values were detected) were replaced by 1/5 of the positive minimum values of the corresponding variables. To improve the results, the data were statistically filtered for variables that were nearly constant throughout the experimental conditions (variables detected using the interquartile range (IQR) at 40%) [68]. MetaboAnalyst 5.0 was also used for biostatistical analysis, including analysis of variance (ANOVA with false discovery rate (FDR) <5%), principal-component analysis (PCA), a heatmap, and boxplots. Protein functions were determined using the STRING.11.5 database [69].

## 5. Conclusions

Although the pandemic of aggressive COVID-19 seems to be under control, SARS-CoV-2 virus infection continues to be a significant medical problem affecting people around the world. Understanding the different metabolic responses of patients to the presence of this virus could help develop targeted therapies based on targeted immune response support. This study for the first time demonstrates the formation and biological efficacy of adducts of proteins—products of lipid metabolism, such as reactive aldehydes 4-HNE and MDA, as well as 15d-PGJ2 in plasma from surviving and deceased COVID-19 patients, significantly distinguishing them, which may be a predictor of the course of SARS-CoV-2 infection.

## Data Availability

Data are available via ProteomeXchange with the identifier PXD044040.

## Supplementary Materials

The following supporting information can be downloaded at: https://www.mdpi.com/article/10.3390/ijms241814109/s1.
